# Classification of Vertebral Osteomyelitis and Associated Judgment Applied during Post-Mortem Inspection of Swine Carcasses in Portugal

**DOI:** 10.3390/foods9101502

**Published:** 2020-10-20

**Authors:** Madalena Vieira-Pinto, Joana Azevedo, Patrícia Poeta, Isabel Pires, Lüppo Ellebroek, Ricardo Lopes, Manuel Veloso, Lis Alban

**Affiliations:** 1 Department of Veterinary Sciences, University of Trás-os-Montes and Alto Douro (UTAD), 5001-801 Vila Real, Portugal; joana.azevedo090@gmail.com (J.A.); ppoeta@utad.pt (P.P.); ipires@utad.pt (I.P.); 2 Animal and Veterinary Research Centre, UTAD, 5001-801 Vila Real, Portugal; 3 Microbiology and Antibiotic Resistance Team (MicroART), Department of Veterinary Sciences, University of Trás-os-Montes and Alto Douro (UTAD), 5001-801 Vila Real, Portugal; 4 Associated Laboratory for Green Chemistry (LAQV-REQUIMTE), University NOVA of Lisboa, 2829-516 Caparica, Portugal; 5 Unit Meat and Food Hygiene, Department Food Hygiene and Animal Health, Federal Ministry of Food and Agriculture, Wilhelmstrasse 54, 10117 Berlin, Germany; Lueppo.Ellerbroek@bmel.bund.de; 6 Faculty of Veterinary Medicine, University of Lisboa, 1300-477 Lisboa, Portugal; lopes.pinto.ricardo@gmail.com (R.L.); manuel.almeida.veloso@gmail.com (M.V.); 7 Department of Food Safety and Veterinary Issues, Danish Agriculture & Food Council, Agro Food Park 13, DK-8200 Aarhus N, Denmark; lia@lf.dk

**Keywords:** vertebral osteomyelitis, meat inspection, post-mortem, inspection, pigs, lesions

## Abstract

Vertebral osteomyelitis (VO) it is often a suppurative lesion that, in Portugal, represents the main cause of total condemnation of slaughtered finishing pigs. Based on the EU Meat Inspection legislation, meat from generalized VO cases presenting signs of pyemia should be declared unfit for human consumption. For that reason, the main objective of this study is to establish a classification scheme to differentiate between localized and generalized VO cases using macroscopic findings and validate it based on the presence of pyemia. To assist in, a combination of macroscopic characteristics of gross lesions (e.g., presence of pyaemia-related lesions (PRL), acute/chronic characteristics of VO) was used to create a classification scheme to differentiate between localized and generalized VO cases. The scheme was applied to 40 VO cases that had been totally condemned in an undifferentiated way. In those 40 cases, histopathological analysis was used to validate acute/chronic macro-criteria, and microbiological analysis was performed to identify the pyemia cases. From the 40 selected VO cases, 20 were macroscopically classified as chronic and 20 as acute. Cohen’s kappa coefficient (*κ* = 0.80; *p* < 0.001), revealed a substantial agreement between macroscopic and histopathology classification. Microbiological analyses identified 13 pyemia cases (13/40; 32.5%). Among those, 12 were macroscopically classified as acute, this association being highly significant (*p* < 0.001). By using the proposed VO classification scheme, 14 possible cases out of 40 could have been spared from total condemnation. This scheme can be used to harmonize the classification of VO and meat inspection decisions in Portuguese abattoirs. The output would lead to avoidance of unnecessary carcasses condemnation (food waste/economic losses), under an evidence-based approach, without compromising food safety and public health as demanded by the EU Meat Inspection legislation.

## 1. Introduction

By definition, vertebral osteomyelitis (VO) is a vertebrae inflammation with medullar cavity involvement [1]. It is often a disfiguring process characterized by necrosis and bone removal leading to compensatory production of new bone; the two processes often occurring simultaneously over a prolonged period [1]. Osteomyelitis is most often secondary to bacterial infections of skin trauma sites, such as wounds from castration, tail biting, and tail docking [1]. *Trueperella pyogenes* and other pyogenic bacteria, like *Streptococcus* spp. and *Staphylococcus* spp., are common causes of suppurative osteomyelitis in pigs and other farm animals [1,2]. For this reason, systemic infection related to osteomyelitis is commonly called pyemia, which means septicemia due to generalized hematogenous spread of pyogenic bacteria [3]. Vertebral osteomyelitis (VO) is the main cause of total condemnation of slaughtered finishing pigs in Portugal, reported by the Competent Authority [4]. Condemnation decisions taken by official veterinarians should be based on Article 45 (f) of EU Implementing Regulation (EU) 2019/627 [5]. This regulation specifies that meat must be declared as unfit for human consumption if it derives from animals affected by a generalized disease, such as septicemia, pyemia, toxemia, or viraemia [5]. In animals affected with VO, pyemia may be present at the time of slaughter. In these cases, carcasses are declared unfit for human consumption. However, if the pyemia has ended weeks or months before slaughter, it seems as unnecessary to condemn the entire carcass. Therefore, it is a challenge during post-mortem inspection to recognize the lesions that indicate the presence of pyemia (generalized disease) and separate it from lesions indicative of prior pyemia (generalized localized disease).

The literature review showed that there are several different definitions for lesions related to pyemia. For Lindén et al. (2014), pyemia may be detected during post-mortem inspection by the presence of suppurative lesions looking like purulent foci of the size from pinpoint to a few millimeters in parenchymal organs like kidneys, lungs, and spleen [2]. According to Jensen et al. (2017), in acute pyemia non-encapsulated purulent lesions can be found in muscle, lymph nodes, and spleen [3]. Also, in soft tissues like liver and lungs, hemorrhages and peripheral hyperemia may be seen. The existence of harmonized criteria to classify cases as either generalized or local based on macroscopic characteristics of the gross lesions found during post-mortem inspection, could ease the work to apply the correct judgment of VO cases. This criteria could be of extreme importance in Portugal because VO is the main cause of condemnation of slaughtered pigs and many of cases are totally condemned in an undifferentiated way because of the perceived risk related to pyemia. Based on this, and in order to support VO classification and judgment during post-mortem inspection, the main objective of this study was to establish a classification scheme to differentiate between localized and generalized VO cases using macroscopic findings and validate it based on the presence of pyemia.

## 2. Materials and Methods

During 17 weeks in the winter of 2016/17, meat inspection of 211,159 pigs was undertaken in one Portuguese abattoir. During this time period, 152 cases of VO were identified during post-mortem inspection. At the first stage of the study, 100 VO cases were included in a detailed investigation allowing recording of macroscopic characteristics of related gross lesions:

- Number of affected vertebrae; when more than two vertebrae were affected, it was also recorded if they were adjacent or not (separated);
- Presence of pyaemia-related lesions (PRL) which means suppurative lesions in other parts of the carcass and/or respective viscera;
- Presence of “perivertebral abscesses” in adjacent muscles resulting from fistulation of VO;
- Classification of VO in acute or chronic. To classify VO lesions as acute or chronic, the following objective macroscopic characteristics were used:
  - Acute: Shiny and moist lesions with, sometimes, congested areas. Evident bone destruction not circumscribed by adjacent remodeling tissue; presence of fluid purulent exudate;
  - Chronic: Moderate bone destruction entirely circumscribed by remodeling tissue; thickened exudate. No evidence of congested areas.

Afterwards, based on dialogue and opinion with stakeholders on meat inspection (Official Veterinarians with practice in swine meat inspection and Professors of Meat Inspection) on literature review [2,3,6,7] and on former EU Meat Inspection legislation [8], a combination of these macroscopic characteristics of related gross lesions were used to develop a scheme to classify VO as generalized or as localized cases that should lead respectively to total and partial condemnation. In this classification scheme, extensive carcass contamination with purulent exudate was also included as a cause of total contamination.

In the second stage of this study, 40 VO cases, totally condemned in an undifferentiated way, were selected according to researcher’s convenience (availability) and classified according to the now proposed VO classification scheme. All macroscopic characteristics were identified directly looking to the internal and/or external surface of carcasses during post-mortem inspection. It was not possible to observe potential presence of additional internal/deeper injuries, since all these cases were declared unfit for human consumption and were immediately removed from the slaughter line for elimination as category 2 animal by-product [9].

To validate the macroscopic classification into acute or chronic, each VO lesion (*N* = 40) was submitted to a histopathological analysis. For that, a bone column fragment with the VO lesion was cut and stored in a container with 10% neutral-buffered formaldehyde for further evaluation. At laboratory, after decalcification, specimens were routinely processed for histological examination, embedded in paraffin wax, and sectioned at 3 μm and stained with hematoxylin-eosin. Histopathological classification of VO lesions was based on the following criteria:

- Acute—Osseous changes: osteonecrosis; irregular contours and fragmentation of bone trabeculae, with bone sequester formation; intramedullary granulocyte infiltrates and fibrin exudates; reduced or complete lack of hemopoiesis. Soft tissue changes: necrosis; inflammatory infiltrate pattern: neutrophilic granulocyte infiltrate diffuse;
- Chronic—Osseous changes: bone neogenesis, medullary space tissue with granulation tissue formation. Soft tissue changes: granulation tissue formation and fibrosis. Inflammatory infiltrate pattern: lymphocyte/macrophage/plasma cell infiltrate, with a few neutrophilic granulocytes.

Additionally, to assess the presence of pyemia in the 40 carcasses with VO, and hereby support the VO classification scheme and associated judgment, paired samples of VO purulent exudate and muscle (diaphragm) were aseptically collected in sterile containers and sent to the laboratory under refrigerated conditions for further microbiological analysis. The presence of the same type of bacteria in both samples was considered as indicative of pyemia. Also, a sample of diaphragmatic muscle was collected from the adjacent carcass fit for consumption (with no lesions) to be used as controls regarding pyemia as outcome. A total of 40 control samples were analyzed. Based on literature review [10] and on a preliminary study carried out by the research team, it was verified that the etiological agents most commonly associated with VO in pigs were *Trueperella pyogenes, Staphylococcus aureus,* and *Streptococcus* spp. This way, the laboratory protocol was adjusted for the identification of these microorganisms. Briefly, samples from purulent content of VO (*n* = 40) were taken with a sterile loop and plated directly on 5% blood agar, consisting of Blood Agar Base No. 2 (OXOID^TM^) and sterile bovine blood. Muscle samples surface (*n* = 80; 40 cases and 40 control samples) were first decontaminated using a branding iron and subsequently 1 g (approximately) was cut out from the depth of the tissue, using a sterile tweezers, suspended in sterile saline solution (1:10) and homogenized in the Stomacher (90 s), and then streaked onto 5% blood agar, consisting of Blood Agar Base No. 2 (OXOID^TM^) and sterile bovine blood.

All plates were incubated at 37 °C for 24 h or 48 h if no growth was visible. After incubation, plates were evaluated and if different cultures were present (only two cases), one colony (isolated) of each was once again streaked onto 5% blood agar and incubated at 37 °C for 24 h to get pure isolates. Afterwards, isolated organisms from purified plates were identified by Gram staining, catalase, and hydrolysis of bile esculin tests [11]. The type of hemolysis was also recorded.

Gram-positive bacilli, which were catalase negative, with no hydrolysis of bile esculin, and isolated from β–hemolysis colonies were classified as suspicious of *Trueperella pyogenes* and were identified using VITEK^®^2 ANC system in the VITEK2 Compact 15 machine (bioMerieux, Marcy-l’Etoile, France), according to the manufacturer’s protocol.

Gram-positive cocci (Oval shape in chains), which were catalase negative with hydrolysis of bile esculin, were classified as *Streptococcus* spp. [11]. Gram-positive cocci (Round in clusters or tetrads), which were catalase positive, with suspicious colonies in Manitol Salt Agar, OXOID^TM^ (yellow colonies with yellow zones in the media) were classified as suspicious of *Staphylococcus aureus*.

### Statistical Analysis

Using IBM SPSS Statistics^®^ 20 software, Fisher’s exact test was used to assess statistical associations between pyemia and each of the macroscopic characteristics of related gross lesions used to classify VO cases. The level of associations between pyemia and each of the macroscopic characteristics of related gross lesions were considered significant (*p* < 0.05). Additionally, odds ratio was calculated to estimate the strength of association between risk factors (e.g., number of vertebrae affected, acute/chronic) and the outcome (pyaemia).

The Kappa test was used to measure the inter-rater agreement between macroscopic and histopathological classification of VO lesions. The inter-rater agreement between macroscopic and histopathological classification of VO lesions were considered significant (*p* < 0.05).

To analyze the validity of VO classification scheme to detect pyemia cases, sensitivity and specificity were calculated. Moreover, to evaluate how well we can rely on the VO classification scheme regarding the presence of pyemia, positive (PPV) and negative predictive values (NPV) were assessed. To calculate all these values, the following table 2 × 2 was created (Table 1).

Sensitivity (Se) is the proportion of true (Py^+^) positives identified by the VO classification scheme as positive. It gives an indication of the ability of the VO classification scheme to correctly identify those cases with pyemia. The formula used, was: Se = *a*/(*a* + *c*)

Specificity (Sp) is the proportion of true (Py^−^) negatives identified by the VO classification scheme as negative. It gives an indication of the ability of the VO classification scheme to correctly identify those cases without pyemia. The formula used, was: = *d*/(*b* + *d*)

The positive predictive value (PPV) reflected the probability that an animal judged as suffering from pyemia using VO classification scheme (VO^+^) was truly affected by pyemia (Py^+^). The formula used, was: PPV = a/a + b.

Likewise, the negative predictive value (NPV) reflected the probability that a negative case identified using VO classification scheme (VO^−^) was truly not affected by pyemia (Py^−^): NPV = *d*/*c* + *d*.

## 3. Results

From the 211.159 finisher pigs slaughtered during the entire study period, 240 carcasses (0.11%) were totally condemned. From those, all VO cases (*n* = 152) were declared unfit for human consumption, representing 0.08% of the slaughtered pigs and constituting the main cause of total condemnation (152/240; 63%).

**Figure 1:**
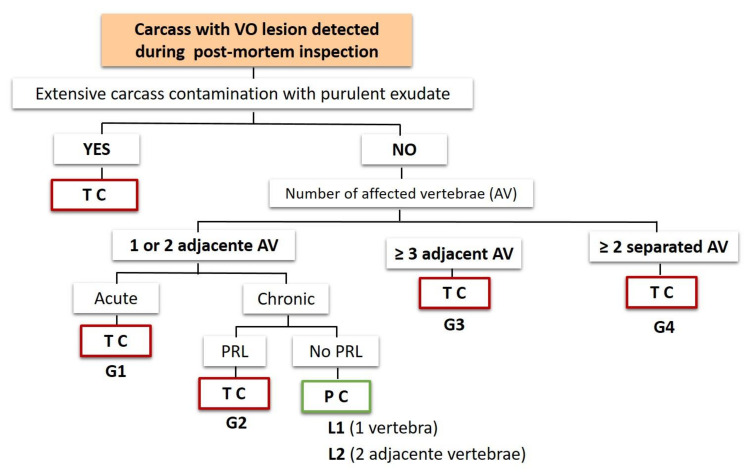
Initial scheme to classify VO cases as generalized (G) or as localized (L) cases. AV—affected vertebrae; PRL—pyemia-related lesions; G1, G2, G3, G4—codes for different types of generalized cases; L1, L2—codes for different types of localized cases; TC—total condemnation; PC—partial condemnation.

In the first stage of this study, using a combination of macroscopic characteristics of related gross lesions, a scheme to classify VO as generalized (G) or as localized (L) was created, based on dialogue and opinion with stakeholders on meat inspection, on literature review [2,3,6,7] and on former EU Meat Inspection legislation [8]. In this scheme, extensive carcass contamination with purulent exudate was also included as a cause of total contamination. Figure 1, schematically presents the now proposed classification scheme.

In the second stage of this study, 40 VO cases were selected according to researcher’s convenience (availability) and classified according to the proposed classification scheme for VO (Figure 1). Overall results are presented in Table 2.

Following the proposed VO classification scheme (Figure 1), all acute cases should be totally condemned, in opposite to chronic cases, in which the judgment depends of the existence of other macroscopic criteria (No. of affected vertebrae, adjacent/separated if two or more vertebrae were affected, PRL presence). Considering the importance of correctly classified macroscopically VO lesions as acute or chronic during post-mortem inspection, validation of macroscopic classification was done based on histopathology as golden standard. Histopathological and macroscopic results are summarized in Table 3.

As presented in Table 3, using the proposed macroscopic criteria, 20 of the 40 VO lesions were classified as acute and 20 as chronic. From those, histopathology analyses identified 18 acute and 22 chronic lesions. Cohen’s kappa coefficient (*κ* = 0.80; *p* < 0.001), revealed a substantial agreement between the two classification procedures (macroscopic/histopathology).

For all the 40 VO cases, microbiological analysis of VO exudate and paired muscle (diaphragm) samples was used to evaluate the presence of pyemia, in order to support the judgment criteria established in the classification scheme proposal (Figure 1). As it was previously described, the presence of the same type of bacteria in both samples (VO exudate and muscle) was taken as the indicative of pyemia. Microbiological results are summarized in Table 4.

Overall results achieved in this study (VO macroscopic characteristics, description of pyemia-related lesions, judgment criteria, and microbiological results) are summarized in Table 5.

To evaluate associations between pyemia and the macroscopic characteristics of related gross lesions (e.g., acute/chronic; number of affected vertebrae, PRL) used to classify VO cases, Fisher’s exact test was performed. From the 13 pyemia cases, 12 were macroscopically classified as acute, this association being highly significant (*p* = 0.0004). Additionally, *odds ratio* result revealed that the odds of pyemia in acute cases is 28.5 times higher as the odds in chronic cases.

Regarding the other macroscopic characteristics (Number of affected vertebrae, adjacent/separated if two or more vertebrae were affected, PRL presence), no significant association with pyemia (*p* > 0.05) was observed.

To calculate sensitivity, specificity, PPV, and NPV values presented in Table 6 were used. For this calculation, only 39 VO cases were considered, since the chronic localized case with extensive contamination with purulent exudate (No. 17, Table 5) was excluded.

Using the scheme proposal for VO classification during post-mortem inspection, the proportion of true (Py^+^) positives cases identified by the VO classification scheme as positive was 100% (Sensitivity). In line, the proportion of true (Py^−^) negatives identified by the VO classification scheme as negative was 42.3% (Specificity).

The values of NPV and PPV were 1 and 0.464, respectively. These values means that the probability of localized cases (VO^−^), that are judge for partial condemned, being truly not affected with pyemia (Py^−^) was of 100% while the probability that a generalized case classified based on VO classification scheme (VO^+^) case is truly affected with pyemia (Py^+^) was of 46.4%.

Taken into account the results found in the second stage of this study, a proposal was made to update the initial classification scheme. In this new proposal, the criterion “number of purulent lesions in different locations” (≥3, 2 or 1) was included, replacing the criterion “Number of affected vertebrae” used in the first scheme. This new criterion includes the number of VO and PRL observed during post-mortem inspection. Regarding the number of affected vertebrae, it was defined that two and three adjacent vertebrae with osteomyelitis was considered as one and two purulent lesion respectively. The following figure shows the new OV classification scheme as well as the number of cases found in this study.

## 4. Discussion

From the 211.159 finisher pigs slaughtered during the entire study period, 240 carcasses (0.11%) were totally condemned. From those, all VO cases (*n* = 152) were declared unfit for human consumption, representing 0.08% of the slaughtered pigs and constituting the main cause of total condemnation (152/240; 63%). These results are in accordance with the ones previously presented by Garcia-Diez et al. (2014) and the Portuguese National Veterinary Authority for the year 2019, reporting VO as the main cause of condemnation, underlining the importance of VO in the pork production chain [4,12].

As it is possible to observe in Figure 1, the initially proposed VO classification scheme was mainly based on whether the osteomyelitis found was judged as acute or as chronic, on the number of affected vertebrae and if they were adjacent or separated, and on the presence pyaemia-related lesions (PRL). PRL was defined as purulent lesions in different tissues than vertebrae, that could be indicative of hematogenous spread of pyogenic bacteria. PRL also included tail lesions that, in this study, were considered as potential source of bacteria entry and dissemination, and hereby the potential cause of osteomyelitis. Also, in the definition of a generalized condition, we included osteomyelitis found in three or more adjacent vertebrae (G3). Although this should not be classified as multiple lesions, the authors consider the hypothesis that this wider lesion may constitute an increased risk for hematogenous spread of pyogenic bacteria (pyaemia). In this classification scheme, localized conditions only included chronic cases affecting one (L1) or two adjacent vertebrae (L2) with no presence of PRL.

Analyzing Table 2, it is possible to observe that, using the classification scheme proposal applied to 40 VO cases, 28 cases (70%, 28/40) were classified as generalized with a sanitary decision of total condemnation. This judgment was also applied to one case (2.5%, 1/40) with extensive carcass contamination with purulent exudate. According to the former EU Meat Inspection legislation (Regulation (EC) No 854/2004 and afterwards Commission Implementing Regulation (EU) 2019/627), meat should be declared unfit for human consumption if shows soiling, fecal, or other contamination [5,8]. The remaining 11 VO cases (27.5%, 11/40) were classified as localized cases that, according to our proposal, could have been spared from the total condemnation.

Regarding the five vertebrae regions (cervical, thoracic, lumbar, sacral, and coccygeal), VO lesions were more commonly observed in only one region (87.5%, 35/40) than in two or more different regions (12.5%, 5/40). From the cases found in only one region, the majority (57.1%, 20/35;) were observed in the thoracic vertebrae compared to the other regions (5 cervical, 4 lumbar, 5 sacral, and 1 coccygeal). These results are not in accordance with the ones presented by Baebko et al. (2016) who observed similar findings for osteomyelitis both in lumbar (17.5%, 21/120) and thoracic (14.2%, 17/120) regions [10].

The affection of a specific vertebral region can be related to surrounding infection tissues, as it may be seen in some coccygeal or sacral osteomyelitis that may occur by direct (local) invasion from a tail lesions [3]. Also, in some cervical osteomyelitis may occur by direct invasion from muscular abscesses that resulted from unhygienic injection procedures. In an opposite way, osteomyelitis in two randomly affected regions may be a signal of hematogenous spread of pyogenic microorganisms [3,7]. Taking this assumption into consideration, the number of affected vertebrae and adjacent/separated characteristics for more than two affected vertebrae were also included in the classification scheme. From the 40 VO cases, the majority only affected one vertebra (55%, 22/40). The remaining 18 cases were observed in two (14) and three vertebrae (4)). From those, lesions were mainly present in adjacent vertebrae (72.2%, 13/18) rather than in separated vertebrae (27.8%, 5/18). In opposite to osteomyelitis in separated vertebrae, this lesion in adjacent vertebrae may be a result a local infection spread from the extension of an intervertebral disc infection or by the contiguity bloodstream [3].

From all the 40 analyzed cases, one that presented osteomyelitis in three contiguous sacral vertebral belonged to a pig data not shown that was found lying at the lairage during ante-mortem inspection (not revealing any signs of septicemia). For that reason, this animal was stunned *in loco* and slaughtered in the first place to avoid unnecessary suffering. Although some authors often considered osteomyelitis as a painful process leading to debilitation of the affected animal [1], in this study only this pig revealed evident clinical signs detectable during ante-mortem inspection. This result suggests that this inspection may not be effective in the detection of VO lesions during ante-mortem inspection.

Additionally to the number and contiguous/separated characteristic of affected vertebrae, the criteria related with the presence of pyaemia-related lesions (PRL) in other parts of the carcass and/or respective viscera beyond VO, may constitute an important parameter to evaluate the presence of a generalized infection [3,6,7].

In the 40 VO cases, the majority (22/40, 55%) did not present additional pyaemia-related lesions observed during post-mortem inspection. In the remaining 18 VO cases (45%, 18/4), PRLs were detected during post-mortem inspection. In addition to joint (7) and tail (3) lesions, muscular abscesses were the main pyaemia-related lesion, being recorded in 66.7% (12/18) of cases with PRL, corresponding to 30% (12/40) of all VO cases. This result is of relevant interest since muscular abscesses mainly occurred as a result of a pyogenic bacteria hematogenous spread [3], and could be a sign for further muscular abscesses. At this point, it is important to remember that, all of the 12 cases with muscular abscesses were identified only looking directly to the internal and/or external surface of carcasses during post-mortem inspection.

Tail suppurative injuries were found in three cases (Table 2). This result represent a surprising lower number since several authors referred tail lesions as an important source of secondary infection leading to pyemia and subsequent vertebral osteomyelitis [1,12,13,14].

It is important to note, however, that although a carcass does not present tail lesions at time of slaughter, it does not invalidate that they have not occurred during the animal’s life and were already healed locally until the time of slaughter and therefore not detected during post-mortem inspection [13,14]. Similar results were also reported by Kruse et al. (2015) [15].

All of these three cases with tail suppurative injury were related to osteomyelitis in thoracic vertebrae. The presence of tail lesions in pigs and pus-containing lesions in the bones (like coccygeal vertebrae) and surrounding tissues can result from local lymph or hematogenous spread (local spread). But, when tail lesions are associated to osteomyelitis lesions in more distant vertebrae (like thoracic), there are different opinions regarding the infection spread from the tail to the vertebrae. Some authors [7] consider that the spreading could occur via the cerebrospinal (not related to pyemia) and others [3] indicating hematogenous spread as a possibility. Based on these results, it is suggested that further studies should be derived to evaluate with more detail the enrolment of tail lesions in VO occurrence.

As it was aforementioned, extensive carcass contamination with suppurative exudate was observed in one case in this study. This contamination was caused not by the VO exudate itself but from purulent content of the respective perivertebral abscesses. Perivertebral abscesses were found in 35 out 40 VO cases (87.5%). According to Weisbrode (2007) and Martínez et al. (2007), perivertebral abscesses occurs due to medulla and bone necrosis with subsequent rupture of the periosteum and the extension of the infection to the adjacent tissue [13,16]. The presence of these abscesses contributes to increasing the spread of purulent exudate during the division of the carcass, leading to its condemnation.

Using the macroscopic criteria proposal to classify the 40 VO lesions into acute or chronic it was possible to identify 20 lesions of each type (Table 2). As it was previously referred by Ninios (2014), it is essential to classify a lesion into acute or chronic during post-mortem inspection in order to properly evaluate if the meat of a slaughtered animal can be accepted or declared unfit for human consumption [6]. Also, Collins and Huey, (2015) and Jensen et al. (2017) underlined the importance to differentiate acute from chronic lesions during meat inspection [3,7].

Considering that acute lesions are more related to generalized disease prior to slaughter (Ninios, 2014) it is important to correctly classify it during post-mortem inspection, avoiding acute false-negatives (classify chronic instead of acute) [6]. In this study, only one case (Table 3) was considered an acute false-negative using the macroscopic classification. Nevertheless, acute classification of VO lesion is not the only variable used to declare a carcass unfit for human consumption. In fact, this false-negative case was judged as unfit for human consumption despite being classified as chronic, presented osteomyelitis in two separated vertebrae and a tail lesion, fulfilling the criterion to be classified as generalized case.

The Kappa test was used in this study to measure the inter-rater agreement between macroscopic and histopathological classification of VO lesions (Table 3). Cohen’s kappa coefficient (*κ* = 0.80; *p* < 0.001) revealed a substantial agreement between the two classification procedures (macroscopic/histopathology). This result allows to indicate VO macroscopic classification in acute and chronic as a reliable proposal to be used during post-mortem inspection, contributing to harmonization of criteria concerning VO classification.

Microbiological results presented in Table 4 showed that *T. pyogenes* was the most frequently isolated from both VO (23) and muscle samples (8), followed by *Streptococcus* spp. (10 VO, 6 muscle) and *S. aureus* (5 VO, 2 muscle). These results are in accordance with the ones previously reported by Bækbo et al. (2016) from deboning finishing pig carcasses with lesions indicative of prior pyemia [10]. No bacteria were isolated from the 40 control muscle samples (Data not shown), which helps to guarantee the confidence on the microbiological procedure, mainly concerning sample contamination.

The presence of bacteria found in VO samples from slaughtered pigs should be seen under two different perspectives. The first one is related to the fact that purulent lesions may harbor significant levels of bacteria that can be a direct source of carcass contamination, especially during carcass splitting step, and an indirect source (cross-contamination) of meat through undue decontaminated equipment and utensils after preparation of affected carcasses. For that, food business operators (FBO) must guarantee the application of adequate decontamination procedures. These practices are associated with additional costs and considerable delays at work, representing extra economic losses to FBO. The second perspective is that *S. aureus*, as well as *T. pyogenes* and *Streptococcus* spp., should be considered as potential occupational risk for slaughterhouse workers, if risk of exposure is not carefully avoided or mitigated [10,17,18].

Concerning to the identified bacteria as potential foodborne pathogen when isolated from the muscle, only *S. aureus* should be considered as a potential risk to consumers. But, for that, carcasses with *S. aureus*, need to reach to the market under conditions (storage at elevated temperatures) that allows the growth of toxigenic cultures responsible for production of enterotoxins level responsible for human intoxication. The bacteria can be killed through heat treatment of the food, but the enterotoxins are very heat resistant, if they have developed as a result of improper handling involving too high temperature. Thus, although the bacteria are eliminated, the toxins may remain and be able to cause disease [18]. According to Kruse et al. (2015), the presence of *S. aureus* should be interpreted with precaution, since these bacteria can also be found in muscle from carcass with no remarks at meat inspection [15]. Meaning that carcasses with VO suppurative exudate containing *S. aureus* may represent an extra source of contamination in slaughtered pigs.

In three cases different bacteria were detected in the paired samples (*T. pyogenes* in VO sample and *Streptococcus* spp. in muscle) indicating that the presence of bacteria in the muscle could be related to another source of infection rather than VO. Interestingly, these three carcasses also presented joint inflammation that can be commonly caused by *Streptococcus* spp., which might help to justify the presence of this bacteria in muscle samples. Nevertheless, according to Bækbo et al. (2016), *Streptococcus* spp. should be considered to be an occupational hazard, rather than foodborne hazard [10].

Overall results achieved in this study: VO macroscopic characteristics, description of pyaemia-related lesions, judgment criteria and microbiological results, are summarized in Table 5. By analyses of this Table, it is possible to see that microbiological analysis identified 13 pyemia cases (13/40; 32.5%). All of the 40 VO cases analyzed in this study are totally condemned. But, according to this microbiological result, only these 13 pyemia cases needed to be totally rejected in order to comply with EU Meat Inspection legislation [5]. Nevertheless, during meat inspection, detection of pyemia it is mainly based on macroscopic analyses to allow recognition of lesions indicative of its presence. For that, associations between pyemia and the macroscopic characteristics of related gross lesions (e.g., acute/chronic; number of affected vertebrae, PRL) used to classify VO cases was assessed using Fisher’s exact test. From the 13 pyemia cases, 12 were macroscopically classified as acute, this association being highly significant (*p* = 0.0004). Additionally, *odds ratio* result revealed that the odds of pyemia in acute cases is 28.5 times higher as the odds in chronic cases. This result highlights “acute characteristic” as important criteria to identify generalized (pyaemia) cases related to vertebral osteomyelitis. This criteria should be taken into account during post-mortem inspection as it was previously described by several authors [3,7]. Also, this result points out the usefulness of the authors’ proposal (evidence-based) for VO classification in acute and chronic lesions. According to Ninios (2014), during meat inspection, classification of acute/chronic lesions is of major relevance, especially in single lesions, since acute single lesions may lead to total condemnation, while chronic single lesions mainly lead to partial condemnation [6]. In this study, pyemia was detected in four single cases (cases No. 7, 14, 16, and 29, Table 5) that presented only VO lesions. In these cases, the use of the VO acute/chronic classification scheme was determinant for application of a proper judgment of total condemnation. In an opposite way, case no. 26 (Table 5), was macroscopically erroneously classified as chronic, since histopathological analyses classified it as acute. In this chronic case, the presence of multiple lesions (osteomyelitis in two separated vertebrae and tail lesions) were determinant to fulfill the criteria to be classified as generalized case, with a judgment of total condemnation.

Although in this study the other macroscopic characteristics (Nº of affected vertebrae, adjacent/separated if two or more vertebrae were affected, PRL presence) used to macroscopically classify VO, revealed no significant association with pyemia (*p* > 0.05), they will be always pointed out as criterion used during post-mortem inspection to support sanitary decision taken by the official veterinarians. Nevertheless, these results, indicates the need to re-evaluate the proposed classification scheme which is based on the risk of pyemia.

To evaluate how well we can rely on the VO classification scheme and respective judgment, it is necessary to look at VO classification as a test. All tests can be characterized by their sensitivity and specificity which are measures of the test’s accuracy. The sensitivity of the VO classification scheme was high (100%), whereas the specificity was lower (42.3%) pointing to an issue with false-positives. However, one could argue that it is more important to look at the predictive values, because those would provide an indication of the usefulness of the test. In the present case, the negative predictive value was 1, implying that when a case is classified as localized using the classification scheme, it is possible to be confident that it is not related to pyemia. In this study, these cases corresponded to 11 chronic VO affecting 1 or 2 adjacent vertebrae with no PRL. Although in these types of cases we can be confident regarding the absence of pyemia, an overall assessment could have been done to evaluate possible dissemination of purulent lesions. As it was aforementioned, abscesses were the main PRL found in this study, advertising for the possibility of existence of other muscular abscesses which should be found. This assessment could be based on the one proposed by Bækbo et al. (2016) [10]. According to these authors to accurately look for the presence of internal abscesses, and in order to make a proper judgement, additional deep cuts should be performed into predilection sites like ham, pork loin, shoulder, and tenderloin [10]. These additional procedures could be done at the rework area, as it was also referred by the same authors [10]. After these procedures, if meat is declared fit for human consumption, the carcass can go for trimming of the affected areas, cutting and deboning if it is hygienically, logistically, and economically realistic. Cutting and deboning processes must occur for aesthetic reasons related to the presence of other abscesses at sites which are not immediately detectable. As it was cited by Collins and Huey, (2015) “There can be nothing more disgusting to the butcher or housewife than to slice through an abscess when preparing meat.” If abscesses are found during cutting and deboning, the procedures in place to handle abscesses should be applied [7].

Moreover, the positive predictive was 46.4% (13/28) which means that, of the 28 generalized VO cases classified during post-mortem inspection, only 13 (28/13; 46.4%) were really affected with pyemia. The remaining 15 cases were wrongly classified as generalized, being false positive considering the presence of pyemia. From those, 8 were classified as acute and 7 as chronic. According to Ninios, (2014), acute cases should lead to total condemnation due to the risk of generalized disease, like pyemia [6]. Also, as it was aforementioned, this study showed a highly significant association between pyemia and acute cases. Based on these facts, those 8 acute cases should be totally condemned.

Since one chronic case (case no. 19, Table 5) that presented three purulent lesions in different locations, revealed the presence of pyemia, we could hypothesize the use of this criterion to classify a generalized case that should lead immediately to total condemnation. Applying this criterion (chronic plus 3 or more purulent lesions) to the 7 chronic false-positive cases, four more cases (case 3, 20, 23, 31, Table 5) were identified with the same characteristics. For this reason, they also should be totally condemned. In an opposite way, the remaining three chronic false-positive cases (case no. 8, 33, 38, Table 5), with two purulent lesions, instead of being immediately total condemned for human consumption, should suffer an overall assessment in the rework area and after, if meat was declared fit for human consumption (depending on the individual case), could go for trimming of the affected areas, cutting and deboning.

**Figure 2:**
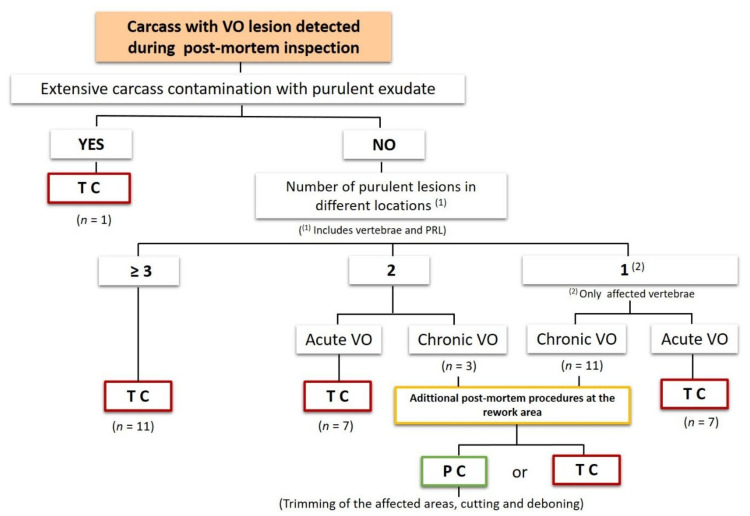
Second proposal of the scheme to classify vertebral osteomyelitis (VO) as generalized or as localized cases that should lead respectively to total and partial condemnation. PRL—pyemia-related lesions; C—chronic; A—acute; TC—total condemnation; PC—partial condemnation.

These results, showed that the criterion “number of purulent lesions in different locations” (number of VO and PRL observed during post-mortem inspection) seems to be of relevance to avoid unnecessary condemnation of other (3) chronic cases in addition to the 11 cases already mentioned. Based on that, a new classification scheme was proposed (Figure 2), where the criterion “number of purulent lesions in different locations” (≥3, 2, or 1) replaced the criterion “Number of affected vertebrae” used in the first scheme.

Using this proposal, a total of 14 cases could have been spared from total condemnation, representing three more cases compared to those spared using the first classification scheme, increasing the specificity (53.8%; 14/26) and PPV (52%; 13/25) of the VO classification scheme. Alternatively, these carcasses, if judged as fit for human consumption after post-mortem inspection at rework area, could go for trimming of the affected parts, cutting and deboning if feasible.

Using this classification scheme, and considering these 14 cases, an economic loss of 1708 € could be avoided. This economic loss is related to the unnecessary condemnation of these carcasses affected by vertebral osteomyelitis considering the carcass weight price 1.6 €/kg, an average carcass weight of 70 kg, and the costs for its elimination as byproduct category 2 of around 10 €/carcass. If we could apply these figures to the national level, where VO is the main cause of condemnation of finishing pigs at slaughterhouse (e.g., 6091 in 2019) [4], a significant amount of euros could be spared every year, highlighting the importance of this study in pork production chain in Portugal.

## 5. Conclusions

In this study, the application of the evidence-based VO classification scheme proposal, proved to be sufficiently effective in eliminating from human consumption the risk cases related to pyemia complying with the objective of the EU Meat Inspection legislation.

The results found highlighted “acute VO characteristic” as an important criteria to identify generalized (pyaemia) cases, points out the usefulness of the authors’ proposal for VO classification in acute and chronic that should now be used by Official Veterinarians during post-mortem inspection.

Using the VO classification scheme proposal, 14 carcasses could have been spared from total condemnation. Nevertheless, since only 46.8% (PPV = 0.468) of the generalized cases, which led to total condemnation, were truly affected with pyemia, more additional studies should be developed in order to understand in what circumstances these cases could be effectively spared from total condemnation, enhancing the economic benefits.

The use of the VO classification scheme during post-mortem inspection of pigs may contribute to the harmonization of criteria concerning VO classification and respective judgment, reducing food waste and economic losses for industry, without compromising food safety and public health.

## Figures and Tables

**Table 1:** Table used to calculate sensitivity, specificity, positive predictive values (PPV), and negative predictive values (NPV), based on VO (Vertebral osteomyelitis) classification scheme (VO) and on pyemia (Py) results.

VO Classification Scheme	True Pyemia Status (Microbiological Analysis)	
	Py^+^	Py^−^
VO^+^ (Generalized)	a	b
VO^−^ (Localized)	c	d

**Table 2:** Results of the classification scheme application to the 40 VO cases analyzed in the second stage of this study.

VO Classification	*n*	%	Number of Affected Vertebrae (AV)	*n*	%
Generalized	28	70% (28/40)	One affected vertebrae (V1)	22	55% (22/40)
G1	20	71.4% (20/28)	More than two AV	18	35% (18/40)
G2	5	17.0% (5/28)	Adjacent AV	13	72.2% (13/18)
			V2	11	84.6% (11/13)
G3	2	7.1% (2/28)	V3	2	15.4% (2/13)
G4	1	3.6% (1/28)	Separated AV	5	27.8% (5/18)
			V2	3	60% (3/5)
Localized	11	27.5% (11/40)	V3	2	40% (2/5)
L1	8	72.7% (8/11)	** Pyemia ** **related lesions (PRL)**		
L2	3	27.3% (3/11)	No PRL lesion	22	55% (22/40)
Extensive carcass contamination with pus	1	2.5% (1/40)	PRL lesion	18	45% (18/40)
**Region of affected vertebrae**			Joint lesion	7	38.9 % (7/18)
One region	35	87.5% (35/40)	Tail lesion	3	16.7% (3/18)
Cervical (C)	5	14.3% (5/35)	Muscular abscesses	12	66.7% (12/18)
Thoracic (T)	20	57.1% (20/35)	1 Muscular abscess	5	41.7% (5/12)
Lumbar (L)	4	11.4% (4/35)	1 Muscular abscess with other lesion * (1 tail *, 1 joint *)	2	16.7% (2/12)
Sacral (S)	5	14.3 % (5/35)	2 Muscular abscesses	3	25% (3/12)
Coccygeal (Co)	1	2.9% (1/35)			
Two different regions	5	12.5% (5/40)	2 Muscular abscesses with other lesion * (1 tail*, 1 joint *)	2	16.7% (2/12)
C+S	1	20% (1/5)	** Perivertebral abscesses **		
T+C	2	40 % (2/5)	No perivertebral abscesses	5	12.5% (5/40)
T+L	2	40% (2/5)	Perivertebral abscesses	35	87.5% (35/40)
**Acute/Chronic classification**					
Acute	20	50% (20/40)			
Chronic	20	50% (20/40)			

VO—vertebral osteomyelitis; G1, G2, G3, G4—codes for different types of generalized cases; L1, L2 —codes for different types of localized cases; vertebrae regions: cervical (C), thoracic (T), lumbar (L), sacral (S), and coccygeal (Co); V1, V2, V3—one, two, and three affected vertebrae with osteomyelitis, respectively. * These lesions are accounted for in the respective group of tail and joint lesions described above. Because some cases had more than one type of PRL lesion, the sum of the relative percentages of each lesion PRL (joint lesion, tail lesion, muscular abscesses) exceeds 100%.

**Table 3:** Results from histopathology and macroscopic analysis of vertebral osteomyelitis cases.

Histopathological Classification	Macroscopic Classification		Total
	Acute	Chronic	
Acute	17	1	18
Chronic	3	19	22
Total	20	20	40

**Table 4:** Microbiological results from vertebral osteomyelitis (VO) and muscle samples.

VO Sample	Muscle Samples				Total
	No Bacteria	*T. pyogenes*	*Streptococcus* spp.	*S. aureus*	
No bacteria	2 (C)	0	0	0	2
*T. pyogenes*	12 (3A, 9C)	8 (7A, 1C)	3 (C)	0	23
*Streptococcus* spp.	7 (3A, 4C)	0	3(A)	0	10
*S. aureus*	3 (2A, 1C)	0	0	2 (A)	5
Total	24 (8A, 16C)	8 (7A, 1C)	6 (3A, 3C)	2 (A)	40

*T. pyogenes*—*Trueperella pyogenes*, *S. aureus*—*Staphylococcus aureus*; A—acute; C—chronic; grey cells, represent pyemia cases (presence of the same type of bacteria in both samples).

**Table 5:** Overall results achieved in this study: VO macroscopic characteristics, description of pyaemia-related lesions, judgment criteria, and microbiological results.

Judgement	Sample No.	VO Classification Generalized (G) Localized (L)		VO Lesion				Pyaemia-Related Lesions (PRL)	*n*	Microbiology			
				Region	No. of AV	A/C				Pyemia * (N)	Only VO (N)	Only Muscle (N)	Bacteria
						M	HP						
**Total condemnation**	1	G1	Generalized cases	Co	V1	A	A	One muscular abscess	1	1	0	0	*T. pyogenes*
	10	G1		C	V1	A	A	One muscular abscess	1	1	0	0	*S. aureus*
	12	G1		S	V1	A	C	Two muscular abscesses	1	0	1	0	*S. aureus*
	6	G1		S	V1	A	A	Joint injury	1	1	0	0	*Streptococcus* spp.
	30	G1		T	V1	A	A	1 muscular abscess and tail injury	1	1	0	0	*T. pyogenes*
	7	G1		C	V1	A	A	No lesions	1	1	0	0	*Streptococcus* spp.
	34	G1		L	V1	A	A	No lesions	1	0	1	0	*T. pyogenes*
	4	G1		S	V1	A	A	No lesions	1	0	1	0	*T. pyogenes*
	36	G1		L	V1	A	A	No lesions	1	0	1	0	*T. pyogenes*
	28	G1		T	V2 adjacent	A	A	1 muscular abscess	1	0	1	0	*Streptococcus* spp.
	40	G1		T	V2 adjacent	A	A	2 muscular abscesses	1	0	1	0	*S. aureus*
	18	G1		C	V2 adjacent	A	A	Joint injury	1	1	0	0	*Streptococcus* spp.
	21	G1		T	V2 adjacent	A	A	Joint injury	1	0	1	0	*Streptococcus* spp.
	14	G1		T	V2 adjacent	A	C	No lesions	1	1	0	0	*T. pyogenes*
	16, 29	G1		T	V2 adjacent	A	A	No lesions	2	2	0	0	*T. pyogenes*
	19	G1		T+L	V2 separated	A	C	Joint injury	1	0	1	0	*Streptococcus* spp.
	11	G1		T+L	V2 separated	A	A	No lesions	1	1	0	0	*T. pyogenes*
	15	G1		T	V3 adjacent	A	A	2 muscular abscesses	1	1	0	0	*T. pyogenes*
	37	G1		C+S	V3 separated	A	A	No lesions	1	1	0	0	*S. aureus*
	33	G2		S	V1	C	C	1 muscular abscess	1	0	1	0	*Streptococcus* spp.
	38	G2		T	V1	C	C	1 muscular abscess	1	0	1	0	*T. pyogenes*
	31	G2		T	V1	C	C	2 muscular abscesses and joint injury	1	0	1	1	*T. pyogenes* (VO) *Streptococcus* spp. (muscle)
	20	G2		T	V1	C	C	1 abscess and joint injury	1	0	1	1	*T. pyogenes* (VO) *Streptococcus* spp. (muscle)
	3	G2		T	V2 adjacent	C	C	2 muscular abscesses and tail injury	1	0	1	0	*S. aureus*
	26	G3		T+C	V2 separated	C	A	Tail injury	1	1	0	0	*T. pyogenes*
	23	G3		T+C	V3 separated	C	C	Joint injury	1	0	1	1	*T. pyogenes* (VO) *Streptococcus* spp. (muscle)
	8	G4		S	V3 adjacent	C	C	No lesion	1	0	1	0	*Streptococcus* spp.
	**Subtotal generalized cases**								**28**	**13**	**15**	**3**	-
	17	**Contamination with pus**		T	V1	C	C	No lesions	**1**	**0**	**1**	**0**	*Streptococcus* spp.
**Partial condemnation**	5	L1	**Localized cases**	T	V1	C	C	No lesions	1	0	0	0	n.i.
	9	L1		T	V1	C	C	No lesions	1	0	0	0	n.i.
	2, 13	L1		T	V1	C	C	No lesions	2	0	2	2	*T. pyogenes*
	22, 24	L1		C	V1	C	C	No lesions	2	0	2	2	*T. pyogenes*
	25, 35	L1		L	V1	C	C	No lesions	2	0	2	2	*T. pyogenes*
	39, 32	L2		T	V2 adjacent	C	C	No lesions	2	0	2	0	*T. pyogenes*
	27	L2		T	V2 adjacent	C	C	No lesions	1	0	1	0	*Streptococcus* spp.
	**Subtotal localized cases**								**11**	**0**	**10**	**0**	-
	**TOTAL**								**40**	**13**	**25**	**3**	**-**

VO—vertebral osteomyelitis; G1, G2, G3, G4—codes for different types of generalized cases; L1, L2—codes for different types of localized cases; vertebrae regions: cervical (C), thoracic (T), lumbar (L), sacral (S), and coccygeal (Co); V1, V2, V3—one, two, and three affected vertebrae with osteomyelitis, respectively; AV—affected vertebrae; A/C—acute/chronic. M—macroscopic analyses; HP—histopathological analyses; * Pyemia—same type of bacteria in both paired samples (VO and muscle).

**Table 6:** Association between VO classification scheme results and true pyemia status of 39 finishing pigs detected with vertebral osteomyelitis during ordinary meat inspection in one Portuguese abattoir in the winter of 2016–2017.

VO Classification Scheme	True Pyemia Status * (Microbiological Analysis)		Total
	Py^+^	Py^−^	
VO + (Generalized)	13	15	28
VO ^−^ (Localized)	0	11	11
Total	13	26	39

* The true status was determined based upon the presence of the same type of bacteria in both samples (VO exudate and muscle).
